# Sclerosing Cholangitis in Behçet's Disease

**DOI:** 10.1155/2013/692980

**Published:** 2013-09-30

**Authors:** Aida Ben Slama Trabelsi, Dhilel Issaoui, Mehdi Ksiaa, Ahlem Souguir, Nadia Mama, Ahlem Brahem, Ali Jmaa, Salem Ajmi

**Affiliations:** ^1^Department of Gastroenterology, Sahloul Hospital, 4000 Sousse, Tunisia; ^2^Department of Radiology, Sahloul, Sousse, Tunisia

## Abstract

*Introduction*. Sclerosing cholangitis is characterized by an inflammatory and fibrotic lesion of intra- and/or extrahepatic bile ducts. When a causal mechanism of a bile duct lesion is identified, the sclerosing cholangitis is considered secondary. The vasculitis, including the Behçet disease, is cited as a probable cause of the ischemia and the sclerosing cholangitis. No cases of extrahepatic secondary sclerosing cholangitis have been reported to date. *Case Report*. We report the first case of secondary sclerosing cholangitis of the extrahepatic bile ducts associated with Behçet disease in a male who is aged 43, with a previous history of the angio-Behçet followed by complications of thrombophlebitis and a cerebral thrombophlebitis, and who has a cholestatic jaundice. The diagnosis has been carried out by the MR cholangiopancreatography which has objectified a moderate distension of the intrahepatic bile ducts upstream of regular stacked parietal thickening of the main bile duct. The patient has been treated successfully with the ursodeoxycholic acid and the placement of a plastic stent. *Conclusion*. This diagnosis should be mentioned to any patient with vasculitis and who has a cholestatic jaundice.

## 1. Introduction 

Sclerosing cholangitis (SC) is characterized by an inflammatory and fibrotic lesion of intra- and/or extrahepatic bile ducts. When a causal mechanism of a bile duct lesion is identified, the SC is considered secondary. The causes of the secondary cholangitis are diverse. Vasculitis, including the Behçet disease (BD), is cited as probable cause of the biliary ischemia and the secondary sclerosing cholangitis (SSC) [1]. Behçet's disease, sometimes called Behçet's syndrome, is a rare immune-mediated small-vessel systemic vasculitis that often presents with mucous membrane ulceration and ocular problems. Behçet's disease (BD) was named in 1937 after the Turkish dermatologist Hulusi Behçet, who first described the triple-symptom complex of recurrent oral aphthous ulcers, genital ulcers, and uveitis. As a systemic disease, it can also involve visceral organs and can be fatal due to ruptured vascular aneurysms or severe neurological complications [2]. An exhaustive review of the literature has found one case of cholestasis referring to an attack of intrahepatic bile ducts for a patient following up a BD [3].

As to our knowledge, we report the first case of secondary sclerosing cholangitis of the extrahepatic bile ducts associated with Behçet disease. 

## 2. Case Description

We report a case of a male who was aged 43, with a previous history of the angio-Behçet with recurrent thrombophlebitis. No other comorbidities was noted.

This patient was hospitalized to explore a cholestatic jaundice installed 15 days before without fever or abdominal pain or alteration of the general state. 

Routine laboratory test found a cytolysis as four times as the normal, gamma-glutamyltransferases with 636 IU/L (*N* < 50 IU/L), alkaline phosphatases with 321 IU/L (*N* = 32–91 IU/L), a total bilirubinemia with 123 *μ*mol/L, and a direct bilirubinemia with 73 *μ*mol/L. The prothrombin rate was 100%.

The serology for HBV and HCV, the antinuclear, anti-liver/kidney microsome type 1, and antismooth muscle and antimitochondria antibodies were negative. 

**Figure 1:**
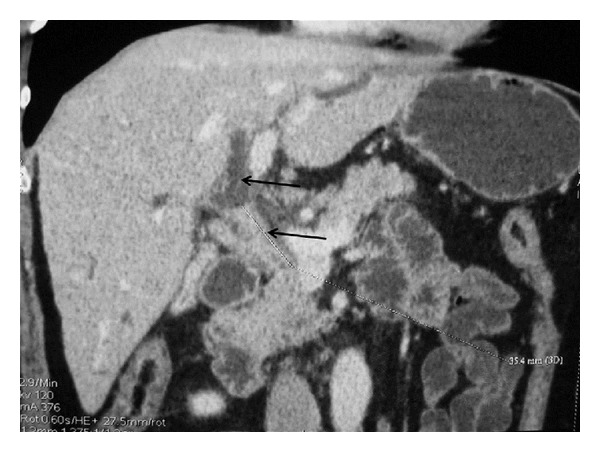
Abdominal CT scan: circumferential thickening of the bile duct with dilatation of the biliary tree upstream.

The abdominal CT scan (Figure 1) showed a peripheral thickening of the main bile duct spreading for 3.5 mm and placed 12 mm away from the roof of the biliary bifurcation, upstream of a distention of the biliary tree. The portal trunk and the extrahepatic veins were permeable.

**Figure 2:**
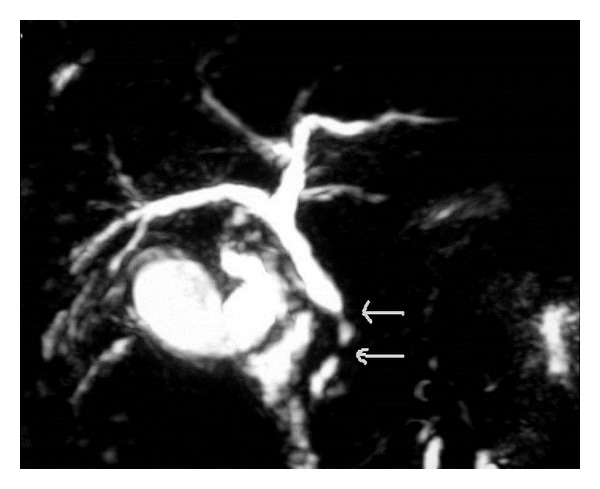
MRI cholangiopancreatography: wall regular thickening of the main bile duct with distal stenoses.

The MR cholangiopancreatography (Figure 2) objectified a moderate distension in the intrahepatic bile ducts with an alternation of the distension and thickening zones of the main bile duct evoking SC lesion. The hepatic biopsy showed intrahepatocyte cholestasis with fibrosis at portal spaces without cholangitis. The diagnosis of a SSC due to the BD was retained because of the regressive cholestatic jaundice, the benign stenoses of the main bile duct, the absence of an existing hepatic pathology and the negativity of the immunological antviral checkup.

The endoscopic retrograde cholangiopancreatography (ERCP), showed stacked stenoses at the level of distal part of main bile duct. The intrahepatic bile ducts were normal. There was a setting up of a plastic prosthesis (diameter 10 french; lengh 8 cm). An ursodeoxycholic-acid-based treatment was initiated with a dose of 25 mg/Kg/j. 

Six months later, the general state of the patient was always conserved, without any jaundice recurrence; the hepatic checkup was normal, and the abdominal CT scan control showed the biliary prosthesis in position without any distension upstream of the bile ducts. 

A second ERCP showed irregular main bile ducts without a distal stenosis. In the fall of this examination, the patient benefited from a removal of the plastic prosthesis without any particular incident. The three years followup showed that the hepatic checkup and the abdominal ultrasonography were normal, and the patient was asymptomatic. 

## 3. Discussion 

The prevalence of the SC is from 10 to 20 per million. Many patients are initially asymptomatic, and the diagnosis is suspected given the presence of abnormality of the hepatic tests. The diagnosis rests on the accumulation of clinical, biological, radiological, and histological arguments [1]. 

A cholangitis is called secondary when the mechanism of the bile duct lesion is determined. 

The causes of SSC are various and are dominated by the prolonged biliary obstructions, the bacterial cholangitis subordinate to a sphincterotomy or to a severe sepsis [4], and the secondary or primitive severe immunodeficiencies [5]. 

The ischemia of the bile ducts has been retained in the cases of SC observed after hepatic transplantation, a biliary surgery [6], an intrahepatic chemotherapy, an arthritis nodosa [7], and a prolonged mechanical ventilation [8]. 

The vasculitis, including the BD, is mentioned as a probable cause of a biliary ischemia and a secondary cholangitis [1]. Actually, an exhaustive review of the literature has found just one case of cholestasis referring to an attack of intrahepatic bile ducts for a patient following up a Behçet disease [3]. For our patient, the diagnosis of an SC of the extrahepatic bile ducts with relation to a lesion of the small vessels of the bile ducts that is secondary to the BD can be retained, due to the fact that it was an angio-Behçet with vascular thromboses and that we eliminated the other possible causes of a SSC. 

The therapeutic ways for the SSC are the same ones that are described during the primitive sclerosing cholangitis (PSC). The ursodeoxycholic acid (UDCA) with a dose of 25 mg/Kg/j is the main therapeutic proposition during the SC. In clinical practice, almost all the PSC (more than 90% in France) are currently getting the UDCA, especially because of its very good tolerance [9]. 

This treatment was used during a secondary cholangitis in a patient at an intensive-care unit with a favorable development [8]. Endoscopic (distension, prosthesis), or more rarely, surgical treatment can be proposed for patients having one stenosis of the extrahepatic bile ducts. A group reported a beneficial effect of the association between the endoscopic distension of stenosis and the UDCA (significant improvement in surviving without any liver transplantation) [10]. In the reported cases of a SSC, the endoscopic or surgery biliary diversion was inefficient in five cases of cholangitis subordinate to a biliary surgery. A hepatic transplantation was indicated in the five cases [6]. 

For our patient, the progress was good under the UDCA and the setting up of a biliary prosthesis. We have observed neither a recurrence of the jaundice nor an appearance of cirrhosis signs under the UDCA up to now. 

## 4. Conclusion 

 We report the first case of secondary sclerosing cholangitis of the extrahepatic bile ducts associated with Behçet disease with a good evolution under medical and endoscopic treatment. 

This diagnosis should be mentioned to any patient with vasculitis and who has developed a cholestatic jaundice.
